# Supplementary material for: KRAS G12V neoantigen specific T cell receptor for adoptive T cell therapy against tumors
Source: Nat Commun. 2023 Oct 12;14:6389. doi: 10.1038/s41467-023-42010-1 (PMC10570350; doi:10.1038/s41467-023-42010-1)
Supplement: Supplementary file 1 — Supplementary Information [file 41467_2023_42010_MOESM1_ESM.pdf]

Supplementary Information

KRAS G12V neoantigen specific T cell receptor for adoptive T cell therapy against tumors

Dan Lu et al.

Supplementary Figures 1-16;

Supplementary Tables 1-6.

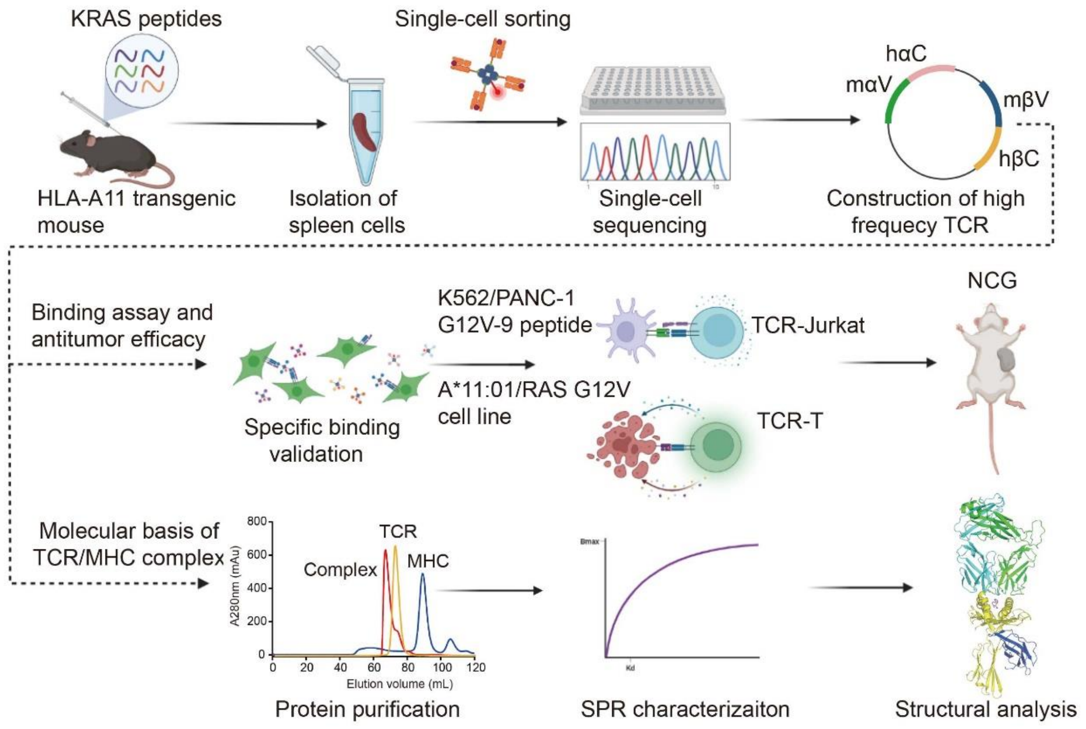

Supplementary Figure 1. Schematic diagram of the technical route of this study.

This diagram was created with BioRender.com.

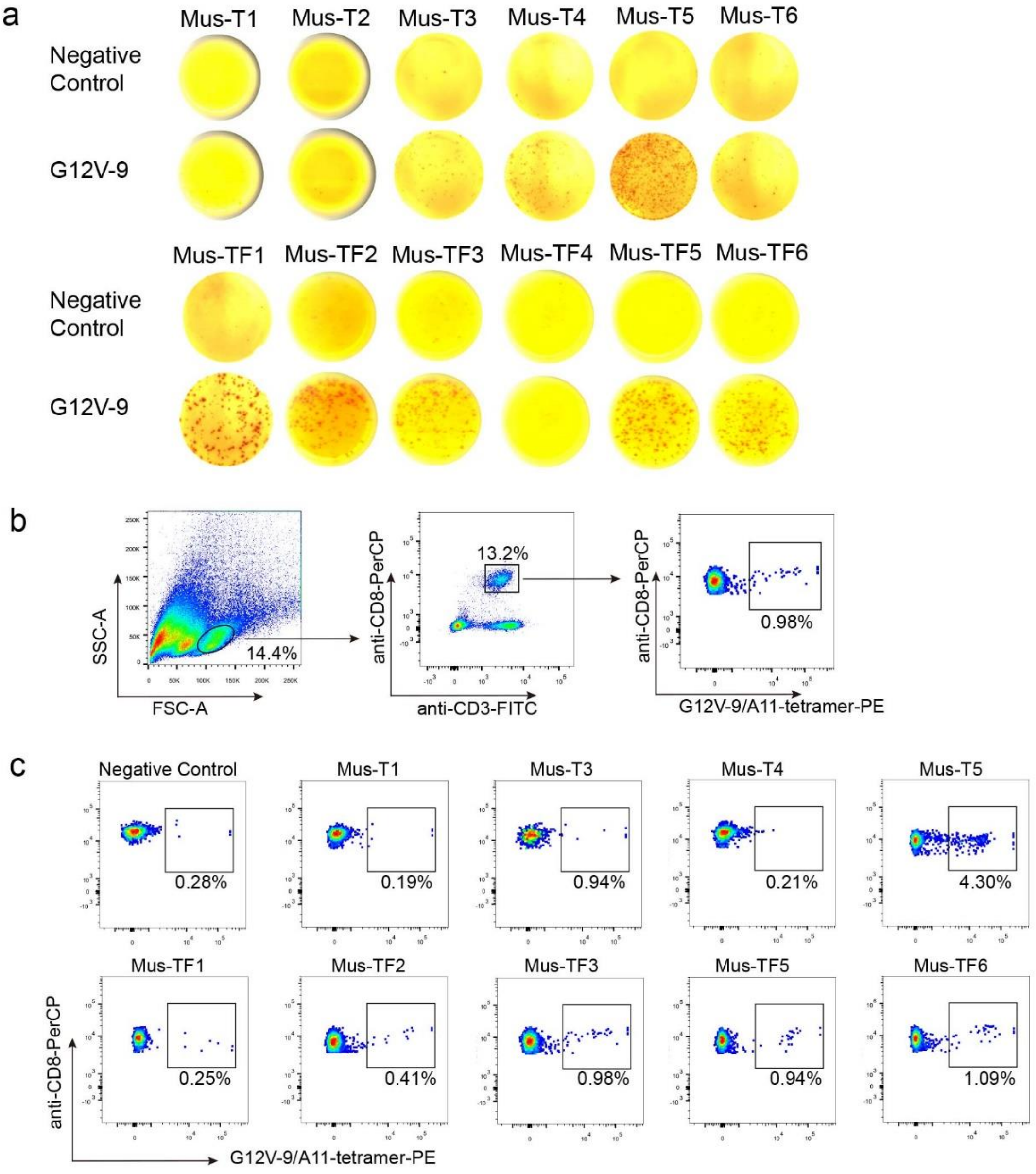

Supplementary Figure 2. Single-cell isolation of KRAS-G12V-9-specific T cells from HLA-A*11:01 transgenic mice.

(a) The immune responses of mouse spleen cells to KRAS-G12V-9 after immunization were evaluated ELISPOT. N=12 of independent immunized mice. (b) Gating strategy for single cell sorting of tetramer specific T cells. (c) Single cell sorting of KRAS-G12V-9/HLA-A*11:01 tetramer^{+} CD8^{+} T cells from mice exhibited positive responses to KRAS-G12V-9 peptide. Mouse without KRAS-G12V-9 peptide immunization was enrolled as the negative control.

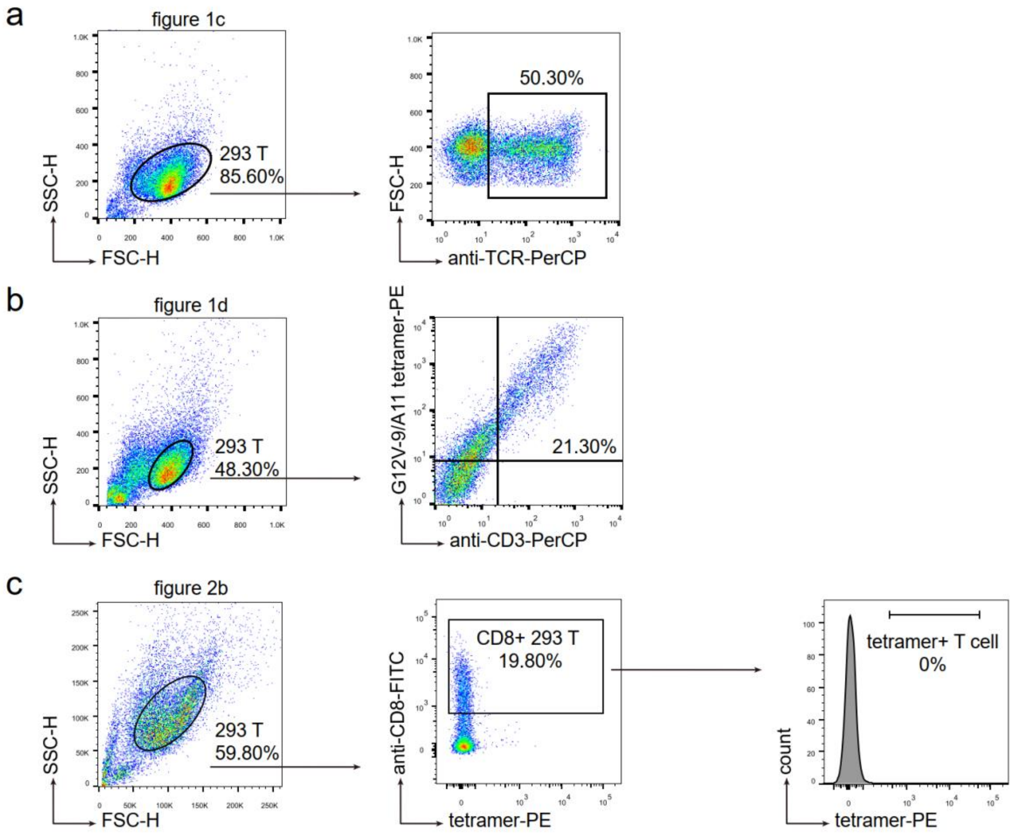

Supplementary Figure 3. Representative flow cytometry gating strategy to detect transfected HEK- 293T cells.

(a) FSC and SSC were used to define the single cell population, the expression of TCR was defined by stained with αβ TCR specific antibody and the positive population was selected. (b) Binding of mutated KRAS-G12V-9/HLA-A*11:01 tetramer with HEK-293T cells transiently expressing indicated TCR was determined by double positivity expression of CD3-PerCP and G12V-9/A11-PE. (c) After gating on singlets, live CD8+ 293T cells were defined on a plot of CD8-FITC vs. tetramer-PE stain, the histogram represents cells stained with tetramer-PE.

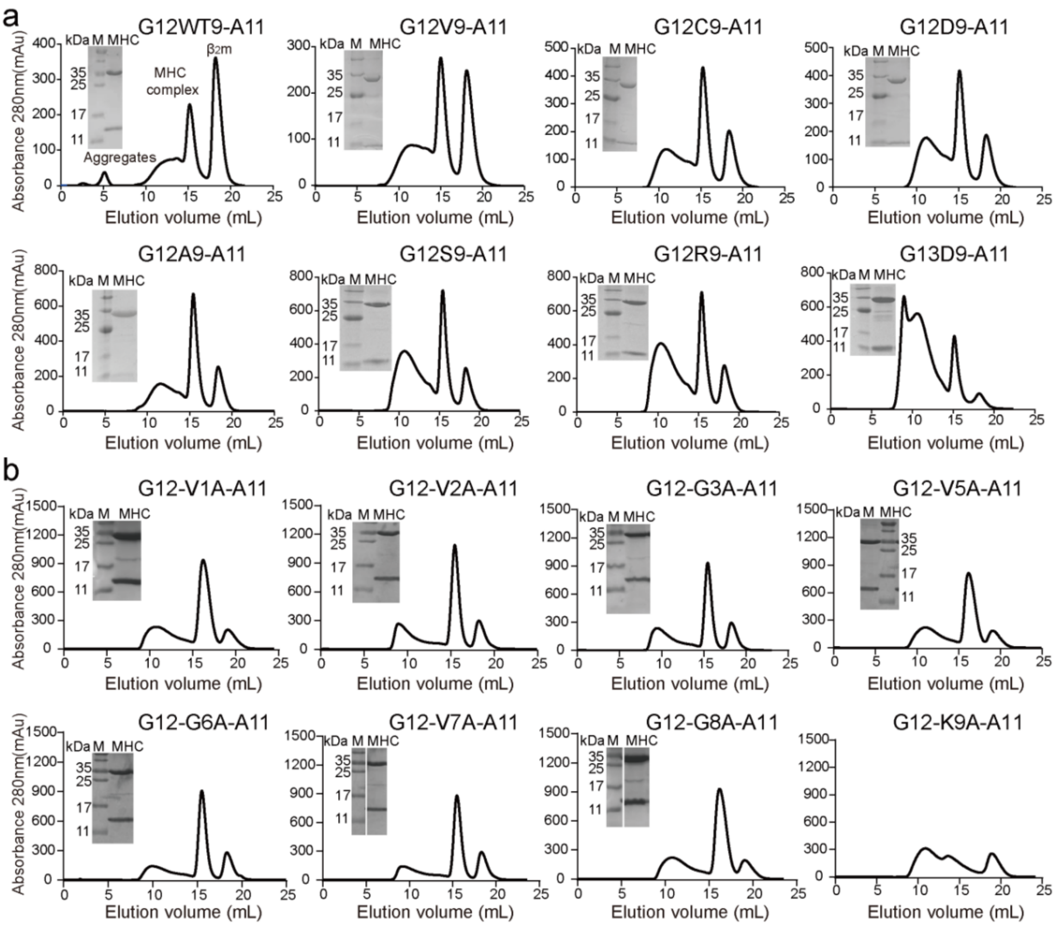

Supplementary Figure 4. The identification of KRAS mutant peptides binding to HLA-A*11:01.

The binding of peptides derived from KRAS mutant neoantigens (a) and alanine-substituted KRAS-G12V-9 peptide (b) with HLA-A*11:01 were evaluated by co-refolding. After properly refolding, the high-absorbance peaks of the correctly refolded MHC-I with the expected molecular mass of 45 kDa were eluted at the estimated volume of 16 mL on a Superdex Increase 200 10/300 GL column. The data shown are from one independent experiment. Source data are provided as a Source Data file.

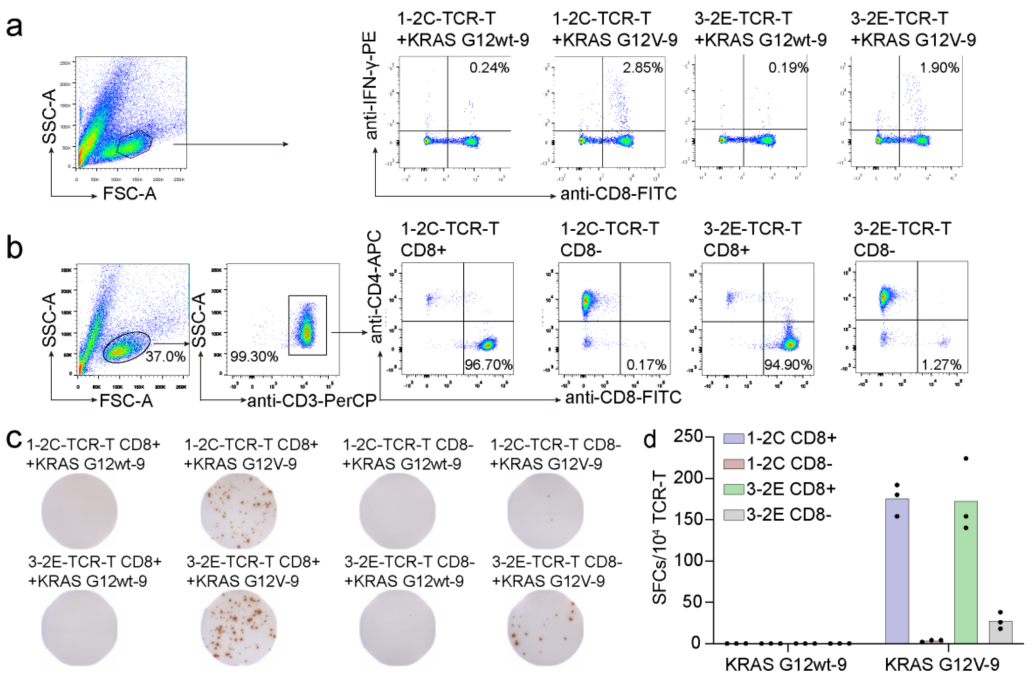

Supplementary Figure 5. CD4/CD8 dependency of 1-2C or 3-2E TCR-T cells upon stimulation with KRAS-G12V-9.

(a) ICS staining of IFN-γ cytokine in 1-2C or 3-2E TCR-T cells stimulated with KRAS-G12wt-9 or KRAS-G12V-9 co-cultured with PANC-1 target cells. The number in the panel represents the percentage of IFN-γ staining positive cells in total T cells. The responses were investigated with 1-2C or 3-2E TCR-T cells prepared with T cells from Donor 1. (b) CD8+ T cells were isolated from 1-2C or 3-2E TCR-T using the CD8+ T Cell Isolation Kit (Miltenyi Biotec, 130-096-495). Cells were fluorescently stained with CD3-PerCP, CD8-FITC and CD4-APC to visualize the target cell fraction. (c) Representative images of CD8+ or CD8- TCR-T peptide-specific spots. The secreted IFN-γ were analyzed by ELISPOT assay with the co-culture supernatants of 1X10^4 TCR-T cells derived from (b) and 2X10^3 PANC-1 cells in the presence of KRAS-G12wt-9 or KRAS-G12V-9. (d) Responses of 1-2C TCR-T CD8+, 1-2C TCR-T CD8-, 3-2E TCR-T CD8+, 3-2E TCR-T CD8- with KRAS-G12V-9. Incubation with KRAS-G12wt-9 served as control. Results are shown as mean of triplicate wells. Data is shown as three technical replicates from two independent experiments. Source data are provided as a Source Data file.

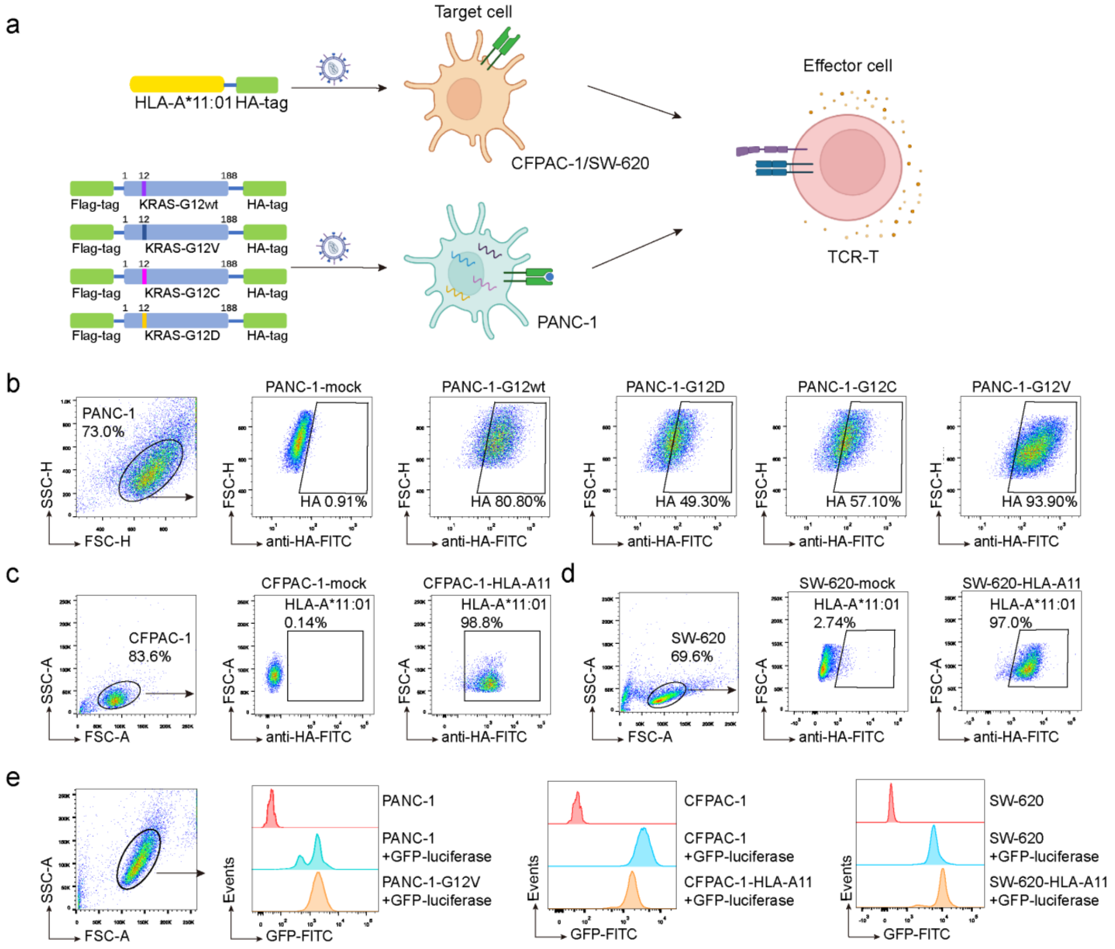

Supplementary Figure 6. Establishment and functional evaluation of HLA-A*11:01/KRAS-G12V target cell line.

(a) Construction strategies of the target cells. CFPAC-1 or SW-620 cells that intrinsically expressed KRAS-G12V mutation were transduced with HLA-A*11:01 through lentiviral transduction, whereas PANC-1 cells intrinsically expressing HLA-A*11:01 were transduced with various KRAS G12 mutants through lentiviral transduction. This diagram was created with BioRender.com. (b-d) Validation of expression efficiency of genetically constructed target cells by staining of specific tags fused to exogenous genes transduced with lentiviruses. (e) The expression of luciferase-transduced cell lines were evaluated by detecting GFP expression by flow cytometry. The data shown are from one independent experiment.

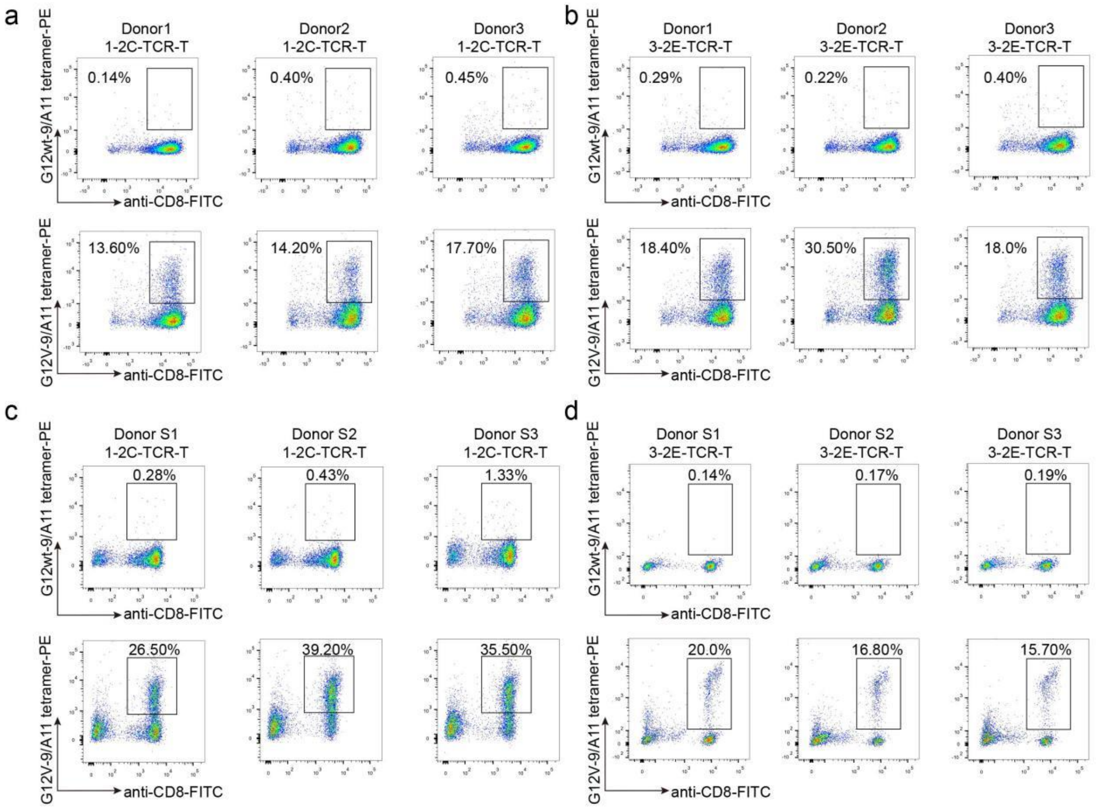

Supplementary Figure 7. Establishment and functional evaluation of TCR-T cells.

1-2C (a, c) or 3-2E (b, d) TCR-T cells engineered with primary T cells from six donors, Donor 1-3, Donor S1-S3, and exogenous TCR expression levels stained with KRAS-G12V-9/HLA-A*11:01 (G12V-9/A11) tetramer, with KRAS-G12wt-9/HLA-A*11:01 (G12wt-9/A11) tetramer as control.

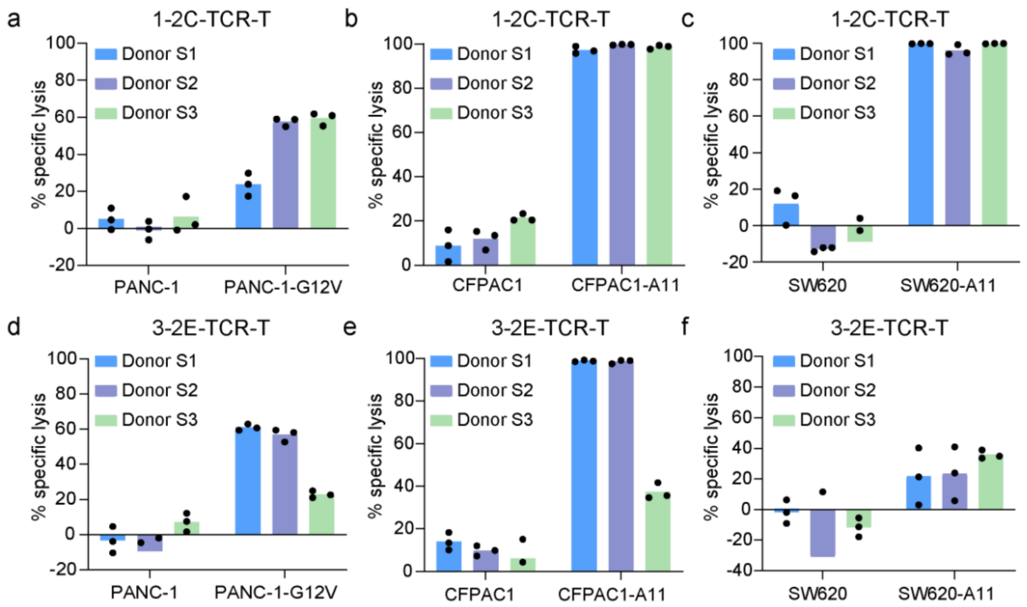

Supplementary Figure 8. Tumor cell lysis measured by luciferase activity in the KRAS-G12V specific tumor cells.

(a) Specific killing of wildtype PANC-1 cells or PANC-1 cells stably expressing mutated *KRAS* genes with G12V site mutation by 1-2C TCR-T. (b, c) Specific killing of wildtype CFPAC-1 cells or CFPAC-1 cells stably expressing HLA-A*11:01 (b), or with wildtype SW620 cells or SW620 cells stably expressing HLA-A*11:01 (c) by 1-2C TCR-T cells. (d-f) Reactivity of 3-2E TCR-T cells against PANC-1, CFPAC-1, and SW-620 as indicated in (d-f). Luciferase-expressing tumor cells (20,000/well) were cocultured with 1-2C/3-2E TCR transduced T cells or untransduced mock-T cells (40,000/well) at an E : T ratio (Effector to Target) of 2 : 1. Luminescence was assessed after 48h later. The dots represent three technical replicates (n=1 experiment) of the responses from three donors (Donor S1-S3). The columns show means of the three technical replicates. 1-2C or 3-2E TCR-T cells prepared with T cells from three donors, Donor S1, Donor S2, and Donor S3. Source data are provided as a Source Data file.

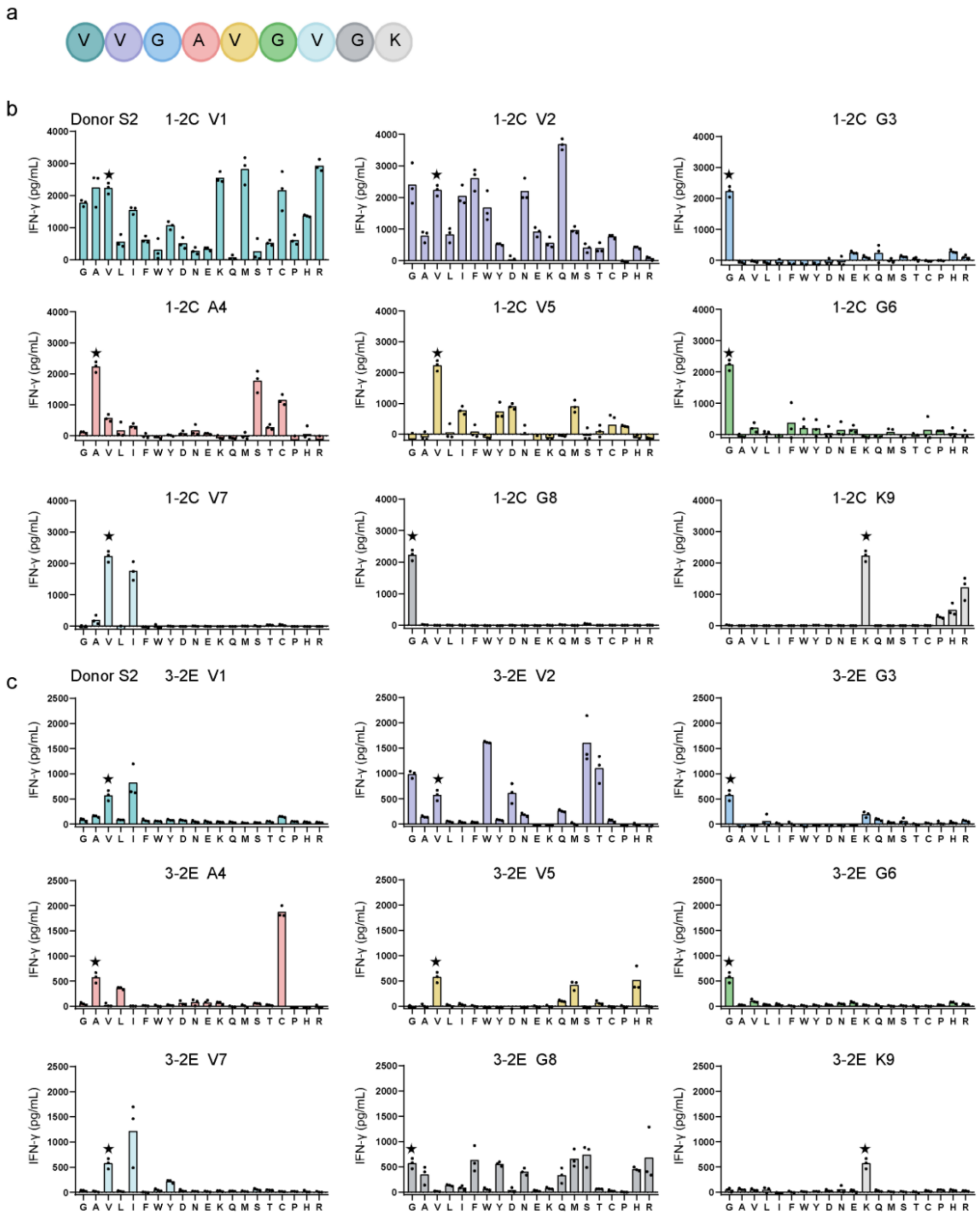

Supplementary Figure 9. Specificity of 1-2C and 3-2E TCR-T cells to combinatorial peptide library screening of KRAS-G12V-9.

(a) Amino acid positions of KRAS-G12V-9. (b, c) 9-mer combinatorial peptide library screen with PANC-1 and KRAS-G12V-9 peptide reactive 1-2C and 3-2E TCR, showing the reactive amino acid residue landscape (KRAS-G12V-9 peptide shown with a black star). Responses of the 1-2C (b) and 3-2E (c) TCR-T cells to combinatorial peptide library. The secreted IFN-γ were analyzed by ELISA assay with the co-culture supernatants of TCR-T cells and PANC-1 cells in the presence of indicated peptides. The responses were investigated with 1-2C or 3-2E TCR-T cells prepared with T cells from another individual donor, Donor S2. The dots represent three technical replicates (n=1 experiment) of the responses from Donor S2. The columns show means of the three technical replicates. Source data are provided as a Source Data file.

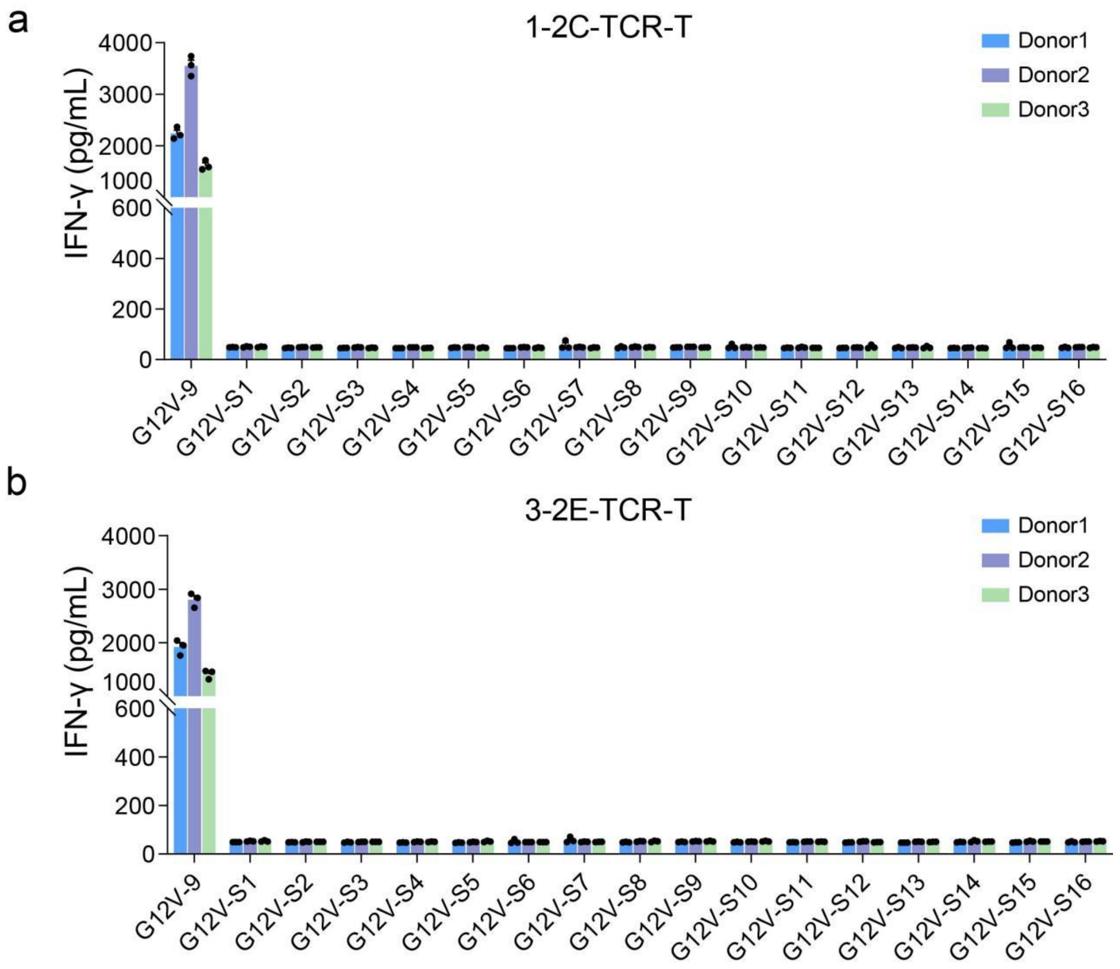

Supplementary Figure 10. Specificity of 1-2C TCR-T cells to homologous peptides in human genome.

Responses of the 1-2C (a) and 3-2E (b) TCR-T cells to homologous peptides, with wildtype and KRAS-G12V-9 peptide tested as negative and positive control. The secreted IFN-γ were analyzed by ELISA assay with the co-culture supernatants of TCR-T cells and PANC-1 cells in the presence of indicated peptides. The responses were investigated with 1-2C or 3-2E TCR-T cells prepared with T cells from three donors, Donor 1, Donor 2 and Donor 3. The dots represent three technical replicates (n=1 experiment) of the responses with T cells from n=3 separate donors (Donor 1-3). The columns show means of the three technical replicates. Source data are provided as a Source Data file.

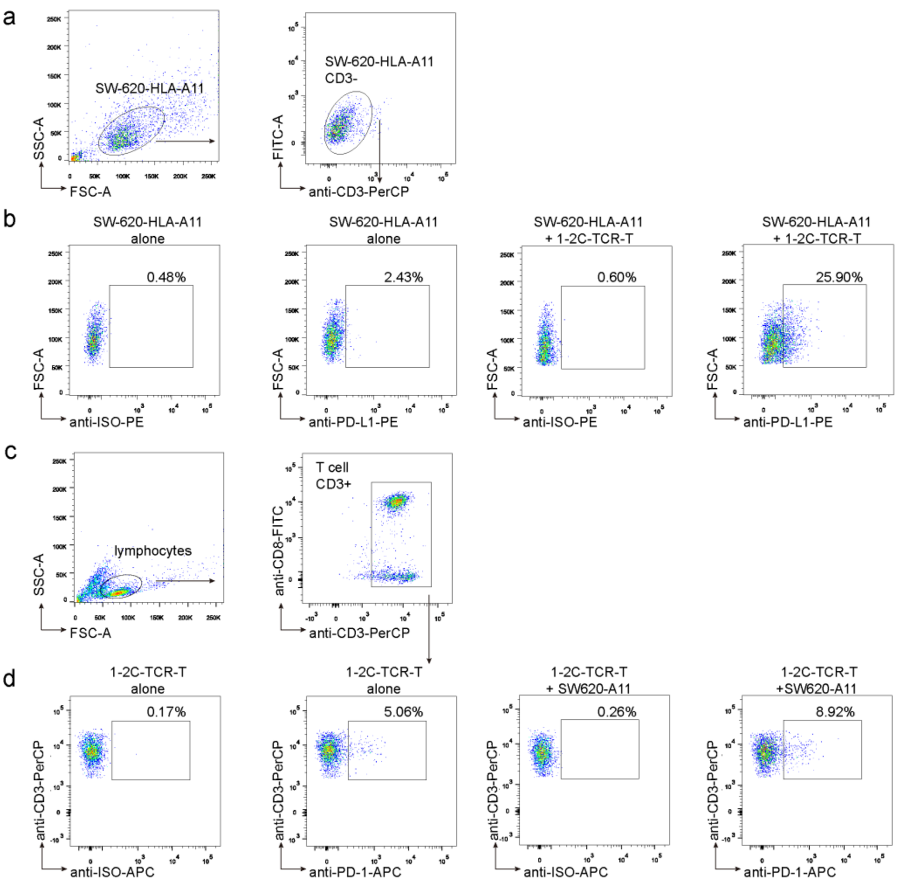

Supplementary Figure 11. The expression of PD-L1/PD-1 detected on the SW-620-HLA-A11 cells and T cells.

(a) Flow gating strategy of PD-L1 expression on SW-620-HLA-A11 cells. (b) PD-L1 expression of tumor cell was measured by flow cytometry in SW-620-HLA-A11 alone and SW-620-HLA-A11 co-cultured with 1-2C-TCR-T 48h, with isotype control. (c) Flow gating strategy of PD-1 expression on 1-2C TCR-T cells. (d) PD-1 expression of 1-2C-TCR-T cell was measured by flow cytometry in 1-2C-TCR-T alone and 1-2C-TCR-T co-cultured with SW-620-HLA-A11 48h, with isotype control. Each experiment was repeated twice in two donors with similar results.

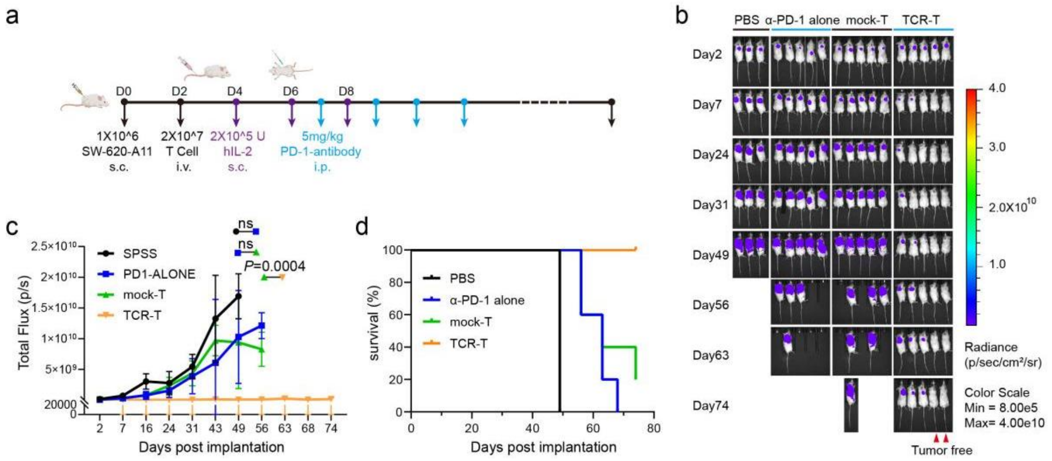

Supplementary Figure 12. Anti-α-PD1 alone treatment did not show substantial any anti-tumor efficacy.

(a) Schematic of the mouse experimental process. (b) Bioluminescence images of orthotopic SW-620-A11 tumor. This diagram was created with BioRender.com. (c) SW-620-A11 tumor progression as evaluated by bioluminescence imaging, n = 3 mice for PBS group and n = 5 mice for the other three groups; data are shown as means ± SD; P values determined by the two-tailed Student's t test and the P values were presented as indicated. ns, P > 0.05. (d) Survival curves of mice bearing SW-620-A11 cells treated with PBS, α-PD-1 alone, mock-T or 1-2C TCR-T cells are shown. The survival curve was generated in GraphPad Prism 10 software. Source data are provided as a Source Data file.

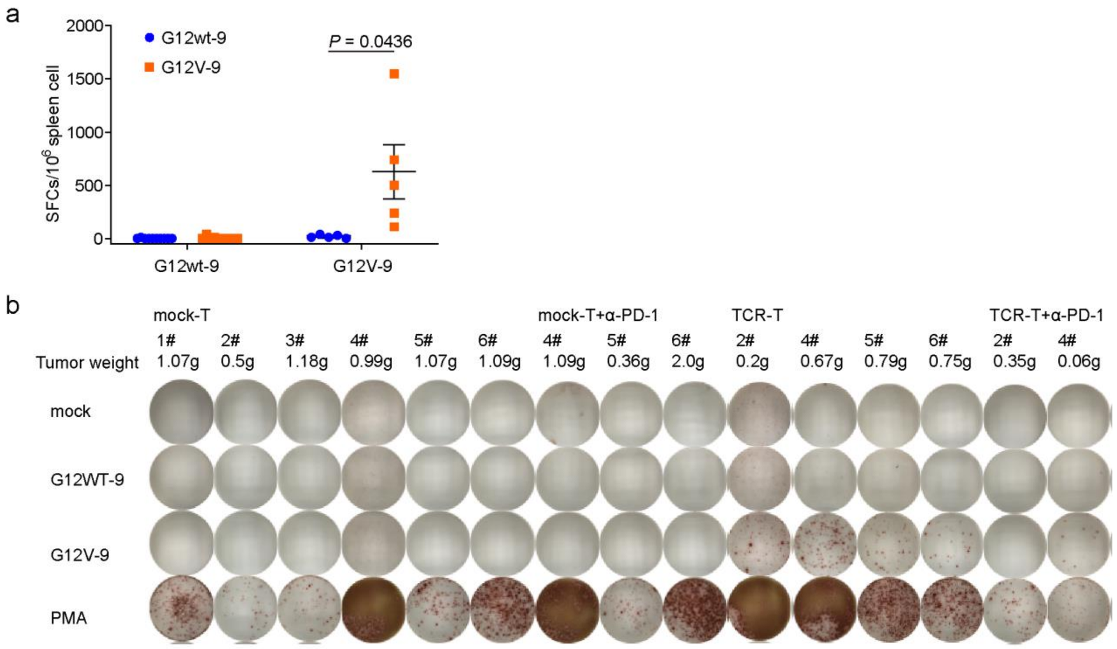

Supplementary Figure 13. The immune response of mouse spleen cells to KRAS-G12V-9 evaluated by ELISPOT.

(a) IFN-γ ELISPOT analysis of the responses of spleen cells in each mouse group stimulated with KRAS-G12wt-9 peptide or KRAS-G12V-9 peptide. The mean responses were shown as black line with the standard deviations represented by the error bars. Statistical analysis was calculated with the two-tailed Student's t test and the P values were presented as indicated. Source data are provided as a Source Data file. (b) Individual reflections for each mouse, not shown for those with too few spleen cells to be tested. The secreted IFN-γ were analyzed by ELISPOT assay with the co-culture supernatants of 1×10^6 spleen cell and 2×10^3 PANC-1 cells in the presence of KRAS-G12wt-9 or KRAS-G12V-9. Spleen cells cultured without peptide were designated as mock control. PMA/ION stimulations were set as the positive control. The data shown are from one independent experiment. The numbers of the mouse in each treatment group were labeled and the responses of each column were from the spleen cells of the same mouse.

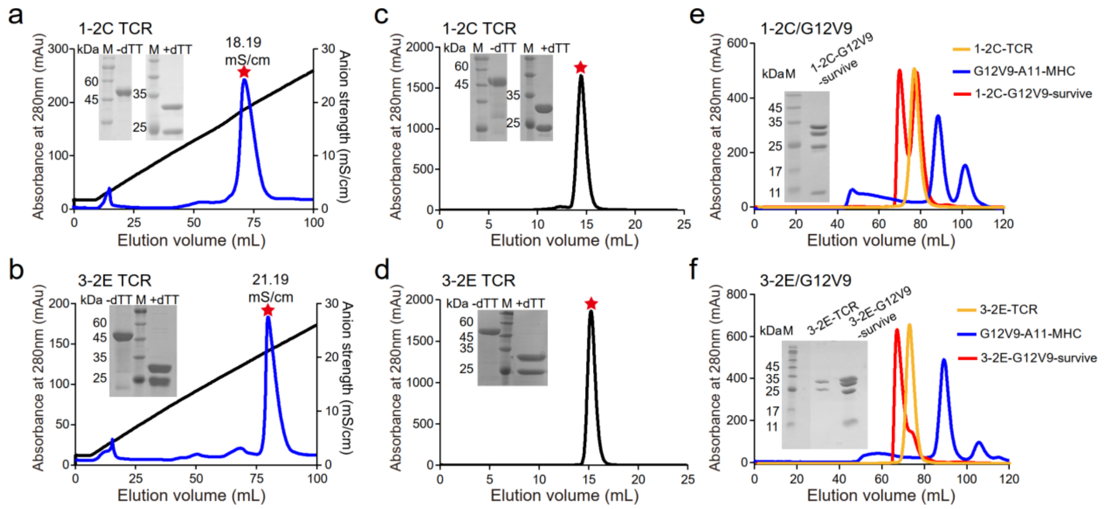

Supplementary Figure 14. Purification of soluble TCRs and pMHCs proteins using a Superdex 200 10/300 GL column.

(a-d) Gel filtration profiles and SDS-PAGE assay confirmation of soluble 1-2C and 3-2E TCR proteins. (e, f) Gel filtration profiles of the 1-2C/HLA-A*11:01-KRAS-G12V-9 and 3-2E/HLA-A*11:01-KRAS-G12V-9 complex were analyzed by size-exclusion chromatography as indicated. The data shown are from one independent experiment. Source data are provided as a Source Data file.

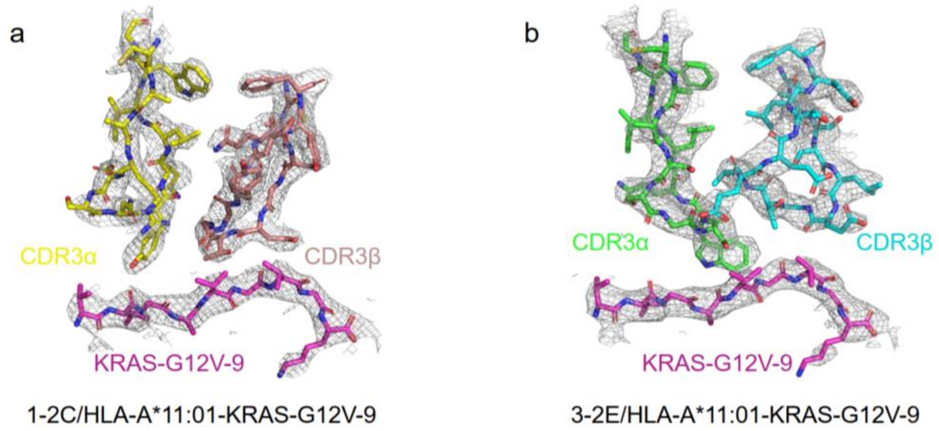

Supplementary Figure 15. Comparison of the two TCR-pHLA complexs and exhibition of electron density maps at the binding interface of TCR and peptides in two complexes.

The electron density maps of binding interface between TCRs and peptides in 1-2C/HLA-A*11:01-KRAS-G12V-9 (a) and 3-2E/HLA-A*11:01-KRAS-G12V-9 (b). The density maps shown in grey 70 from the final 2Fo – Fc map are controlled at 1σ. 1-2C CDR3α, yellow; 1-2C CDR3β, salmon; 3-2E CDR3α, green; 3-2E CDR3β, cyan; KRAS-G12V-9, hotpink.

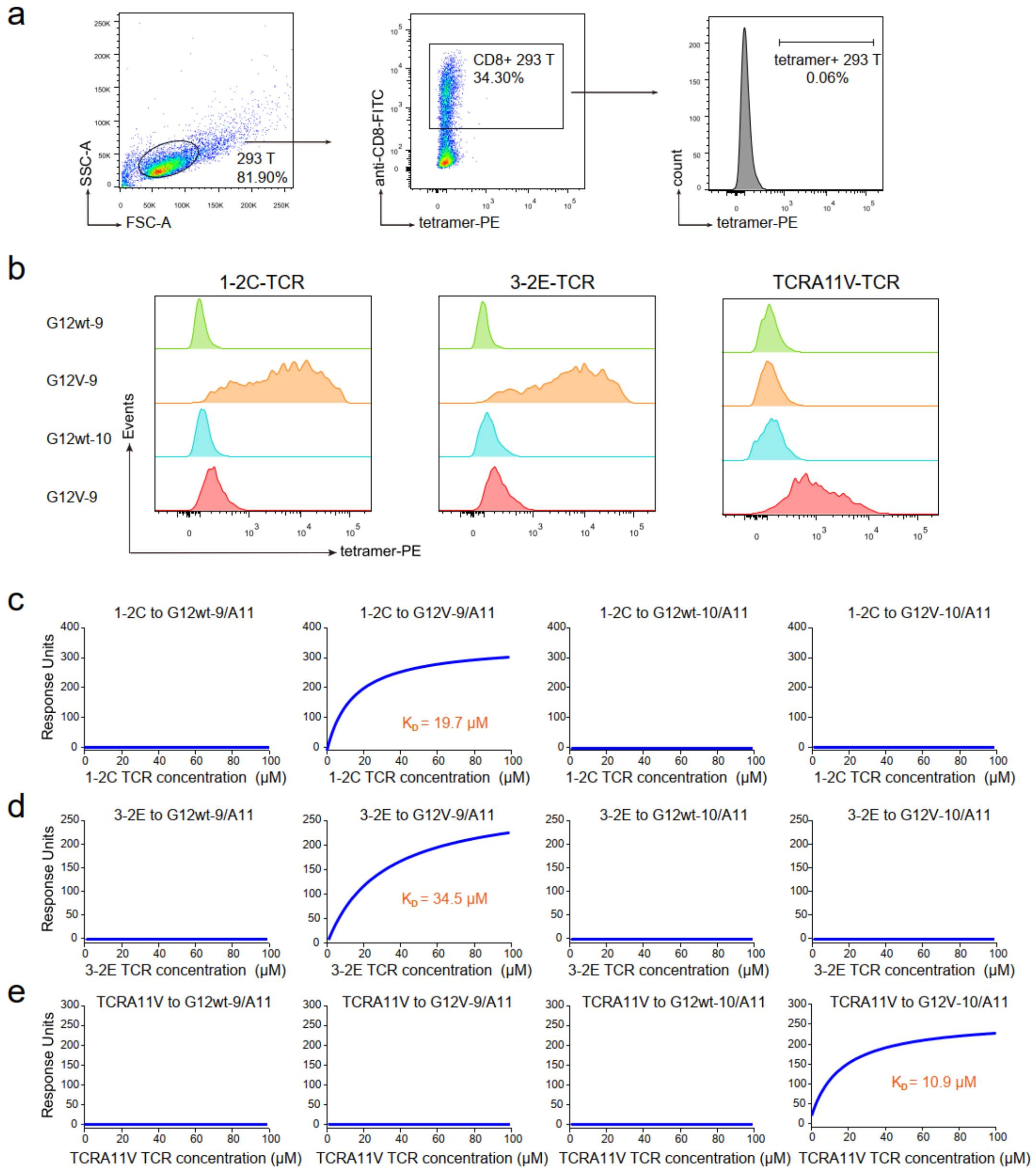

Supplementary Figure 16. Specific binding of 1-2C, 3-2E, and TCRA11V TCRs to 9-mer or 10-mer KRAS-G12V mutant peptide.

(a) Gating strategy used for flow cytometry analysis. (b) Binding of pHLA tetramers loaded with 9-mer or 10-mer wild type or KRAS-G12V mutant peptides to 1-2C, 3-2E or TCRA11V TCR expressed on HEK-293T cells was analyzed by flow cytometry. The data shown are from one of two independent experiments. (c-e) SPR assay characterization of the binding profiles of 1-2C (c), 3-2E (d) and TCRA11V (e) TCR with pHLA proteins loaded with 9-mer or 10-mer wild type or KRAS-G12V mutant peptides. The data shown are from one independent experiment. Source data are provided as a Source Data file.

Supplementary Table 1. Sequence of oligoclonal TCRs identified from KRAS-G12V-9 specific murine splenocytes

ID	Vα	Jα	CDR3α	Vβ	Dβ	Jβ	CDR3β
1-2C	TRAV7-2*02	TRAJ33*01	CAARDSNYQLIW	TRBV13-2*01	TRBJ2-7*01	TRBD1*01	CASGDTGGYEQYF
3-2E	TRAV8-1*03	TRAJ22*01	CAASSGSWQLIF	TRBV16*01	TRBJ2-3*01	TRBD1*01	CASSLEGTVEETLYF
4-1G	TRAV16D/DV11*03	TRAJ44*01	CAMRGFTGSGGKLTL	TRBV17*01	TRBJ2-5*01	TRBD1*01	CASSSRVNQDTQYF
A13B16	TRAV13N-3*01	TRAJ32*01	CAMGWVGSSGNKLIF	TRBV16*01	TRBJ2-3*01	TRBD1*01	CASSLEGTVEETLYF
A13B13	TRAV13-5*01	TRAJ40*01	CVLSGGNYKYVF	TRBV13-2*01	TRBJ2-7*01	TRBD2*01	CASGDTGGYEQYF
A7B13	TRAV7-2*02	TRAJ22*01	CAASSSGSWQLIF	TRBV13-3*01	TRBJ2-5*01	TRBD1*01	CASSDSRGSQDTQYF
A17B12	TRAV17*02	TRAJ57*01	CALEGQNQGGSAKLIF	TRBV12-2*01	TRBJ2-5*01	TRBD2*01	CASSQWGGGQDTQYF

Supplementary Table 2. Peptides used in this study

Peptide name	Amino acid sequence
KRAS-G12wt-9	VVGA**G**GVGK
KRAS-G12V-9	VVGA**V**GVGK
KRAS-G12C-9	VVGA**C**GVGK
KRAS-G12D-9	VVGA**D**GVGK
KRAS-G12A-9	VVGA**A**GVGK
KRAS-G12S-9	VVGA**S**GVGK
KRAS-G12R-9	VVGA**R**GVGK
KRAS-G13D-9	VVGAG**D**VGK
KRAS-G12wt-10	VVVGA**G**GVGK
KRAS-G12V-10	VVVGA**V**GVGK
KRAS-G12C-10	VVVGA**C**GVGK
KRAS-G12D-10	VVVGA**D**GVGK
KRAS-G12V-1A	XVGAVGVGK*
KRAS-G12V-2A	VXGAVGVGK
KRAS-G12V-3A	VVXAVGVGK
KRAS-G12V-4A	VVGXVGVGK
KRAS-G12V-5A	VVGAXGVGK
KRAS-G12V-6A	VVGAVXVGK
KRAS-G12V-7A	VVGAVGXGK
KRAS-G12V-8A	VVGAVGVXK
KRAS-G12V-9A	VVGAVGVGX

*X mean this position was substituted with 20 amino acids.

Supplementary Table 3. Homologous peptides in human genome used in this study

Name	Sequence	Resource	NCBI Reference Sequence
G12V-9	VVGAVGVGK	GTPase KRas isoform b [*Homo sapiens*]	NP_004976.2
G12V-S1	VVGAGGVSK*	carnosine synthase 1 isoform 1 [*Homo sapiens*]	NP_001159694.1
G12V-S2	VVGPVGCGK	ATP-binding cassette sub-family C member 3 isoform 1 [*Homo sapiens*]	NP_003777.2
G12V-S3	VVGGGGVGK	ras-related protein R-Ras [*Homo sapiens*]	NP_006261.1
G12V-S4	IVGRTGAGK	ATP-binding cassette sub-family C member 3 isoform 1 [*Homo sapiens*]	NP_003777.2
G12V-S5	VVGDGGVGK	ras-related protein M-Ras isoform 1 precursor [*Homo sapiens*]	NP_001078518.1
G12V-S6	VVGAVDSGK	tigger transposable element-derived protein 6 [*Homo sapiens*]	NP_112215.1
G12V-S7	VLGAPGVGK	ras-like protein family member 10A [*Homo sapiens*]	NP_006468.1
G12V-S8	VVGQVGCGK	multidrug resistance-associated protein 1 [*Homo sapiens*]	NP_004987.2
G12V-S9	VIGDLGVGK	ras-related protein Rab-38 [*Homo sapiens*]	NP_071732.1
G12V-S10	IVGCTGSGK	dynein heavy chain 2, axonemal isoform 1 [*Homo sapiens*]	NP_065928.2
G12V-S11	VVGNVGFGK	protein TANC1 isoform 1 [*Homo sapiens*]	NP_203752.2
G12V-S12	VVGGLAVGK	H(+)/Cl(-) exchange transporter 7 isoform a [*Homo sapiens*]	NP_001278.1
G12V-S13	VVGNAGTGK	dynein heavy chain 11, axonemal [*Homo sapiens*]	NP_001264044.1
G12V-S14	VIGPVGSGK	ATP-binding cassette sub-family C member 2 [*Homo sapiens*]	NP_000383.2
G12V-S15	VVGRTGAGK	ATP-binding cassette sub-family C member 2 [*Homo sapiens*]	NP_000383.2
G12V-S16	VVGPPGTGK	RNA helicase aquarius [*Homo sapiens*]	NP_055506.1

*The variant residues were highlighted with an underline.

Supplementary Table 4. Contacts of HLA-A*11:01 with 1-2C TCR or 3-2E TCR

1-2C/HLA-A*11:01 KRAS-G12V9				3-2E/HLA-A*11:01 KRAS-G12V9			
Region	1-2C TCR	HLA-A*11	Contacts (H bond）	Region	3-2E TCR	HLA-A*11	Contacts (H bond）
CDR1α	N29	R163	2[a]	CDR1α	T27	R163	1
	D31	R163	14 (2)[b]		S28	R163	7
	Y32	Q155	11 (1)			E166	5 (1)
	W34	Q155	1			W167	2
CDR2α	F51	E154	4		T30	A158	7
		R157	5			Y159	5
		A158	13			R163	1
	K56	E154	1	CDR2α	R50	E154	14 (1)
	R92	Q155	1			E155	8
CDR3α		A158	1			A158	1
		R163	2		S51	A158	5 (1)
	D93	R163	5 (1)			G162	1
	S94	Q62	4 (1)		N52	R157	6 (1)
		R163	4			A158	3
	N95	Q62	3			E161	2
		R65	9 (1)	FR3α	T67	E166	1
		N66	3 (1)	CDR3α	S93	R163	15 (1)
		R163	1		G94	R163	2
	Y96	N66	5		W96	N66	16
		Y159	2			A69	11
		R163	10			Q70	2
	N28	K146	1			T73	3
					Q97	Q62	21
CDR1β	N30	T73	9 (1)	CDR2β	R50	T73	10 (1)
		V76	2		N51	V76	5
	Y48	R65	4		A53	Q72	2
FR2β	Y50	R65	8	FR3β	I55	R65	2
CDR2β		N66	1			A69	3
		A69	12			Q72	2
		Q72	7		D56	Q62	1
	G51	Q72	5			R65	12 (1)
	A52	Q72	3		S58	R65	7
	S54	Q72	3				
	E56	R65	1				
	Q71	V76	2				
FR3β	T96	A69	2				
CDR3β		T73	1	CDR3β	E97	Q62	10
	G97	Q155	3			A150	4

	G98	Q155	12 (1)		T99	Q155	4
	Y99	A150	2		E101	Q155	13 (1)
		H151	23				
		E154	4				
		Q155	14				
Total contacts			224	Total contacts			204

a. Numbers represent the number of atom-to-atom contacts between 1-2C/3-2E TCR and HLA-A*11 residues, which were analyzed by the Contact program in CCP4 suite (the distance cutoff is 4.5 Å).

b. Numbers in the parentheses represent the number of hydrogen bonds between 1-2C/3-2E TCR and HLA-A*11 residues which were analyzed by the Contact program in CCP4 suite (the distance cutoff is 3.5 Å).

Supplementary Table 5. Contacts between HLA-A*11:01 and KRAS-G12V9

KRAS-G12V9	HLA-A*11:01	Contacts	Total
V1	M5	2a	63 (1)
	Y7	14 (1)b	
	Y59	3	
	E63	9	
	Y159	6	
	R163	12	
	W167	12	
	Y171	5	
V2	W7	1	38 (1)
	W9	3	
	M45	1	
	E63	8 (1)	
	N66	5	
	Y99	5	
	Y159	9	
	R163	1	
G3	Y99	9 (1)	34 (2)
	Q156	4 (1)	
	Y159	21	
A4	Y9	4	23 (1)
	N70	4	
	Y99	11 (1)	
	R114	2	
	Q156	2	
V5	R114	7	24 (1)
	Q155	5	
	Q156	12 (1)	

KRAS-G12V9	HLA-A*11:01	Contacts	Total
G6	Q70	2	4
	T73	2	
V7	W147	11	14
	A152	1	
	Q155	2	
G8	T73	1	11
	D74	1	
	D77	7	
	T80	2	
Y9	D77	20 (1)	76 (4)
	T80	3	
	L81	1	
	Y84	5 (1)	
	I95	2	
	I97	3	
	D116	6	
	T143	13 (1)	
	K146	5	
	W147	18 (1)	
Total contacts			287

a. Numbers represent the number of atom-to-atom contacts between 1-2C/3-2E TCR and HLA-A*11 residues, which were analyzed by the Contact program in CCP4 suite (the distance cutoff is 4.5 Å).

b. Numbers in the parentheses represent the number of hydrogen bonds between 1-2C/3-2E TCR and HLA-A*11 residues which were analyzed by the Contact program in CCP4 suite (the distance cutoff is 3.5 Å).

Supplementary Table 6. Contacts of KRAS-G12V9 peptide with 1-2C TCR or 3-2E TCR

1-2C / HLA-A*11:01 KRAS-G12V9				3-2E / HLA-A*11:01 KRAS-G12V9			
Region	1-2C TCR	KRAS-G12V9	Contacts (H bond）	Region	3-2E TCR	KRAS-G12V9	Contacts (H bond）
CDR3α	Y96	V2	5 (1)	CDR3α	S95	V5	7
		G3	3			G6	1
					W96	A4	3
						G6	5
CDR1β	N30	G6	1	CDR2β	R50	G6	6 (2)
CDR3β	D95	G6	8			V7	3
		V7	10			G8	2 (1)
	T96	V5	1	CDR3β	E97	G6	1
		G6	1			V7	1
	G97	V5	5		T99	G6	2
	G98	V5	1			G7	1
total contacts			35	total contacts			32

a. Numbers represent the number of atom-to-atom contacts between 1-2C/3-2E TCR and HLA-A*11 residues, which were analyzed by the Contact program in CCP4 suite (the distance cutoff is 4.5 Å).

b. Numbers in the parentheses represent the number of hydrogen bonds between 1-2C/3-2E TCR and HLA-A*11 residues which were analyzed by the Contact program in CCP4 suite (the distance cutoff is 3.5 Å).
